# Prevalence and risk factors of malaria and anaemia and the impact of preventive methods among pregnant women: A case study at the Akatsi South District in Ghana

**DOI:** 10.1371/journal.pone.0271211

**Published:** 2022-07-25

**Authors:** Asiwome Ahadzie-Soglie, Otchere Addai-Mensah, Albert Abaka-Yawson, Anita Mawuse Setroame, Precious Kwablah Kwadzokpui

**Affiliations:** 1 Department of Medical Laboratory Technology, Faculty of Allied Health Sciences, Kwame Nkrumah University of Science and Technology, Kumasi, Ghana; 2 Laboratory Department of the Ho Teaching Hospital, Ho, Ghana; 3 Department of Medical Diagnostics, Faculty of Allied Health Sciences, Kwame Nkrumah University of Science and Technology, Kumasi, Ghana; 4 Department of Medical Laboratory Sciences, School of Allied Health Sciences, University of Health and Allied Sciences, Ho, Ghana; PLOS: Public Library of Science, UNITED KINGDOM

## Abstract

**Aim:**

This study aimed to ascertain the prevalence and risk factors of malaria and anaemia as well as the impact of preventive methods among pregnant women at the Akatsi South District Hospital of Ghana.

**Subjects and methods:**

A hospital based cross-sectional study using simple random sampling technique was conducted among 200 pregnant women receiving antenatal care and laboratory services at the Akatsi District Hospital from May 2016 to July 2016. A semi-structured questionnaire was administered to obtain participants’ malaria preventive methods in addition to demographic and gestational details. Participants’ hemoglobin and malaria status were assessed using one milliliter (1 ml) whole blood collected from each participant following standard procedures. Factors that produced a p-value of ≤0.2 from the univariate model were included in the final model. Association between potential covariates and the outcomes was assessed using multivariate logistic regression. The Clopper-Pearson test statistic was used to determine the 95% confidence intervals of the outcome variables of interest. We also estimated the population attributable fraction (PAF) of anaemia due to malaria by substituting the adjusted relative risk estimates (RR_*i*_) (using the *adjrr* command in STATA) of anaemia due to malaria into the category-specific attributable formula. P-values of <0.05 were considered statistically significant.

**Results:**

Prevalence of anaemia in pregnancy (AiP), malaria in pregnancy (MiP) and AiP/MiP comorbidity was 63.5% (95% CI:56.4–70.2), 11.0% (96% CI:7.0–16.2) and 10.5% (95% CI:6.6–15.6) respectively. Prevalence rates of AiP (66.7%) and MiP (18.5%) predominated among pregnant women aged < 20 years. PAF of AiP due to MiP was 34.5% (95% CI:23.8–43.6). High use of IPTp-SP, 64.0% (95% CI:56.9–70.6) and LLIN, 90.0% (95% CI:85.0–93.8) was observed in this study. Only 42.0% (95% CI:35.1–49.2) used repellent. Not being on the IPTp-SP program posed a 11.70 times risk of MiP (95% CI:2.32–58.96; p = 0.003) compared to pregnant women on the IPTp-SP program. Similarly, not sleeping under LLIN posed an 8.07 times risk of MiP (95% CI:1.98–32.2; p = 0.004) compared to pregnant women who slept under LLIN. Meanwhile, being positive for MiP posed a 12.10 times risk (95% CI:1.35–85.06; p = 0.025) of AiP compared to those negative for malaria whereas failure to attend ANC as scheduled posed 6.34 times risk (95% CI:1.81–22.19; p = 0.004) of AiP among the pregnant women studied.

**Conclusion:**

The prevalence of MiP and AiP among pregnant women in the Akatsi South District remains a great concern. High utilization of IPTp-SP and LLIN was observed with a resultant positive effect on malaria prevalence among pregnant women. Improved access to IPTp-SP and LLIN is hence encouraged to help further diminish the risk of malaria infection amongst pregnant women in the District.

## Introduction

Malaria has in several decades been identified as a devastating public health problem particularly among pregnant women in the tropics [1, 2]. The acute fibril illness is caused by five different Plasmodium species namely *P*. *falciparum*, *P*. *vivax*, *P*. *malariae*, *P*. *knowlesi* and *P*. *ovale* [3] however, *P*. *falciparum* accounts for the majority of malaria cases in pregnant women [1]. According to the WHO 2020 World Malaria Report, an estimated 229 million malaria cases (95% CI; 211–252 million) and 405,000 deaths (95% CI; 387,000–460,000) was recorded globally in 2019 while in the WHO African Regions alone, an estimated 215 million cases (95% CI; 197–237 million) and 384,000 (95% CI; 365,000–433,000) deaths was reported same year. In its report, the WHO African Region accounted for about 94% of the malaria cases and deaths globally [4]. In sub-Saharan Africa, MiP remain a significant public health problem with an estimated 25 million pregnant women living at risk of *P*. *falciparum* malaria infection [3]. Significant burden if infected includes annual low birth weight (LBW) babies because of a combination of preterm delivery (PTD) and fetal growth restriction (FGR), abortion of the fetus or death of pregnant woman in severe cases, stillbirth and impairment of fetal development [5–7].

The most devastating and life-threatening effect of malaria in pregnancy is the development of anaemia characterized by a reduction in the number of red blood cells, often accompanied by reduced hemoglobin levels or altered RBC morphology [8]. Anaemia prevalence of 5% or higher in a population study is classified as public health significance according to the WHO. If a population study documents anaemia prevalence rate of greater than or equal to 40%, that prevalence is classified as a severe public health problem [7, 9]. Anaemia is an associated cause of increased morbidity and mortality in women and children, poor birth outcomes, impaired cognitive and behavioral development in children and declined work productivity in adults [10]. Prevalence of anaemia according to findings is highest among pregnant women in Sub-Saharan Africa (SSA) (57%), followed by pregnant women in Southeast Asia (48%), and lowest (24.1%) in South America [9]. In Ghana, a multicenter study of 628 pregnant women revealed an overall anaemia prevalence of 42.4% representing a significant public health problem. Prevalence of mild, moderate and severe anaemia was 35.7%, 6.1% and 0.6% respectively with malaria being an independent risk factor [7]. A significant reduction of malaria among the population will therefore correspond to a significant reduction in anaemia cases.

The fight in combating malaria dates as far back as the 1950s when Ghana’s health system at the time adopted the Indoor Residual Spraying (IRS) against adult mosquitoes, weekly swallowing of daraprim (referred to as “Sunday Sunday”), mass chemoprophylaxis with pyrimethamine medicated salt and basic sanitation practices to improve drainage systems. Decades on, the continuous distribution of long-lasting insecticide treated bed nets (LLIN), seasonal malaria chemoprevention (SMC) and rapid diagnostic testing (RDT) at health facilities were introduced [11, 12]. Ghana’s current malaria prevention strategy includes prompt and effective case management, sleeping under insecticide-treated nets especially the long-lasting ones and taking medication for intermittent preventive treatment at monthly intervals after 16 weeks [13]. These interventions are in line with the WHO’s recommendation [7]. According to the 2019 Malaria Indicator Survey, 43% of the household population, 54% of children under age 5, and 49% of pregnant women slept under an LLIN the night before the survey. Eighty percent of pregnant women received at least two doses of IPTp-SP for the prevention of malaria in pregnancy, and 61% received at least three doses [14]. The use of LLINs have been indicated to diminish maternal malaria parasitemia by 38%, malaria-related anaemia by 47%, low birth weight by 23%, miscarriages/stillbirths by 33% and placental parasitemia by 23% [7].

In the Akatsi South District, malaria consistently remained the topmost disease among the top ten diseases that affect the majority of its population. Despite recording a reduction in the prevalence rate since 2014 to 2017 [2014 (40.64%); 2015 (20.00%); 2016 (30.03%); 2017 (22.9%)], the infection has remained the major concern of the people of Akatsi South District [15]. The consistency in the incidence of malaria prompted the district to embarked on several programs such as distribution of LLIN, mass spraying of drains and gutters and malaria sensitization programs to curb the menace [15]. Currently, there is paucity of data on the impact of MiP preventive methods such as IPTp-SP and LLIN utilization in the Akatsi South District of Ghana. This study therefore assessed the prevalence and associated risk factors of malaria and anaemia and the impact of IPTp-SP, LLIN and mosquito utilization among pregnant women in the Akatsi South District, Ghana.

## Materials and methods

### Study design and eligibility criteria

**Fig 1:**
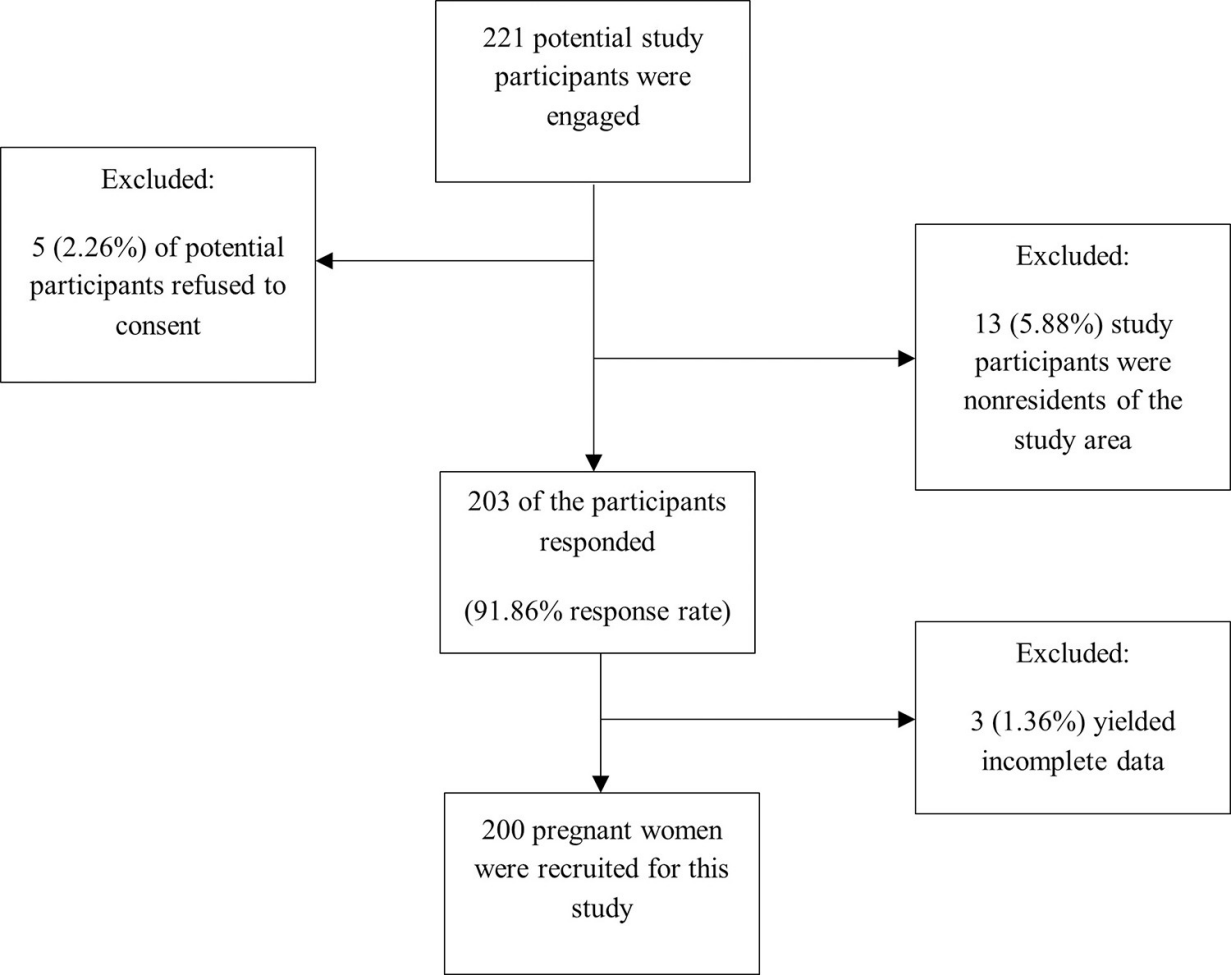
Flow diagram of recruited pregnant women included in the study.

A hospital based cross-sectional study using simple random sampling technique was carried out among 200 pregnant women (Fig 1) receiving antenatal care and laboratory services at the Akatsi South District Hospital, Ghana from May to July, 2016. The Akatsi District hospital is one of the 29 health facilities in the District (Source: Akatsi South District Health Directorate, 2017). The hospital at the time of this study had a staff strength of 140 healthcare workers including two doctors, a medical assistant, a pharmacist, and a biomedical scientist. The hospital is a 70-bed capacity facility, with a maternity unit, medical laboratory unit, family planning unit and an outpatient department. It also serves as a referral center for the surrounding primary health facilities in the district. The choice of Akatsi South District for this study emanates from the fact that malaria consistently remained the topmost disease among the top ten diseases that affect the majority of its population [15]. Pregnant women who were registered with the Akatsi South District Hospital but were not residents of the Akatsi South District were excluded from the study to ensure that reported prevalence rates of malaria and anaemia represent an accurate reflection of the true status of malaria and anaemia prevalence among participants from the study area only. Non-consenting pregnant women were also excluded from the study. Participation of the respondents was voluntary.

### Sample size

The minimum sample size for the study was determined using the Raosoft Online Sample Size Calculation formula (http://www.raosoft.com/samplesize.html) with the following parameters; 5% margin of error (E), 95% confidence level, average annual antenatal attendance (N) of approximately 2500, a response rate (r) of 85% (obtained from a prior pilot study), number of ‘positive’ observations (x) of 4898.04 and a critical value (*Z*(c/100)) of 1.96.

The required formulas are:

$$x=Z\;{\left(\frac{c}{100}\right)}^{2}r(100-r)$$ (1)

$$n=\frac{Nx}{\left((N-1)E^{2}+x\right)}$$ (2)

Let Z(c/100) = 1.96

r = 85%

N = 7796

Hence x = 1.96^2^ * 85(100−85) = 4898.04

Substituting N = 2500 and x = 4898.04 into (2) to determine the minimum sample size n, gives

$$n=\frac{2500*4898.04}{\left((2500-1)5^{2}+4898.04\right)}=181.7507=182$$

Hence, the minimum required sample size for this study was 182 pregnant women from the Akatsi South District. Two hundred (200) pregnant women was however sampled to increase statistical power and reliability of the findings.

### Data collection

A semi-structured questionnaire was administered to the pregnant women by the researchers. The questionnaire was designed in English and then translated via interview to the respondent in the language best understood by the respondent: Ewe and Twi predominantly.

The sociodemographic characteristics of the respondents were maternal age, level of education, occupation, location, residential status, and marital status.

Obstetric details included gravidity, parity, the gestational period in weeks (definition: **first trimester**, from week 1 to the end of week 12; **second trimester**, from week 13 to the end of week 26 and **third trimester**, from week 27 to the end of the pregnancy) and attended ANC as scheduled.

Malaria preventive methods were IPTp-SP use, sleeping under LLIN and the use of mosquito repellent.

None of the respondents reported using insecticide sprays, mosquito coils, and/or creams to prevent malaria in this study.

### Laboratory investigations

#### Blood film for malaria parasites

About one milliliter (1 ml) whole blood was collected from each pregnant woman from the median cubital vein of the antecubital fossa of the arm into an EDTA tube using a sterile syringe and needle following standard blood collection protocols. Samples were collected at the onset of the rains, between May and July 2016 which marks the period of the malaria transmission season. The blood film preparation and observation were done following standard procedures outlined by Monica Chesbrough [16]. Briefly, thick blood films were then prepared on a clean grease-free glass slide using 6 ul of whole blood, spread evenly to cover a dimension of about 15x15 mm of the center of the glass. The smears were air-dried, fixed in absolute methanol, and then left to air-dry in about 1–2 minutes ready for staining. The slides were then stained with 10% Giemsa for 10 minutes and then examined under oil immersion (magnification x100) with a light microscope. Reading of the slides was carried out by two trained microscopists. The absence of malaria parasite after 200 high power fields was examined was considered negative for malaria. A discrepancy in the slide reading outcome between the two initial scientists was resolved by a third opinion from a senior microscopist.

#### Hemoglobin estimation

Participants’ hemoglobin concentration was determined using the fully automated Sysmex XS-800i hematology analyzer (https://www.sysmex-europe.com/n/products/products-detail/xs-800i.html; Europe). All test procedures were carried out following standard quality-controlled procedures. Anaemia in pregnant women was defined as a hemoglobin concentration less than 11.0 g/dL while hemoglobin concentration greater than or equal to 11.0 g/dL was considered normal. Anaemia was further subclassified as mild (hemoglobin: 10–10.9 g/dL), moderate (hemoglobin: 7–9.9 g/dL) and severe anaemia (hemoglobin <7 g/dL) [17].

#### Quality control steps

Our Sysmex XS-800i hematology analyzer was quality controlled following the manufacturer’s instruction by running manufacturer’s provided quality controlled samples to ensure the accuracy and precision of the instrument before running the samples of the study participants. Known malaria positive and negative slides were used to quality control the 10% Giemsa stain. Two independent and qualified parasitologists examined 15% of both positive and negative well prepared randomly selected slides. A third and final opinion of a third senior parasitologist was sought in the instance of any discrepancy in the two initial examinations. Consistency in microscopic outcomes signaled good working Giemsa stain.

### Data handling and analysis

Data collected from the questionnaires and results from the laboratory investigations were checked for consistency, completeness and then captured into Microsoft Excel 2016 using fit-for-purpose excel form to avoid as many as possible entry errors. Data were then exported into Statistical Package for Social Sciences (SPSS) for Windows (version 21.0) and STATA (version 15.0) where appropriate for statistical analysis. All data were categorical, therefore, descriptive analysis was done and presented as frequencies and percentages in parenthesis. Chi-square and Fisher’s exact test where appropriate were used to assess statistical associations between explanatory variables and outcome variables. Factors that produced a p-value ≤0.2 from the bivariate model were included in the final model. Association between potential covariates and the outcome variables was assessed using multivariate logistic regression. Explanatory variables having a p-value <0.05 from the multivariable model were considered as having a statistically significant association with the outcome. Measures of association were determined using adjusted odds ratio with a 95% confidence interval (CI) from the multivariate logistics regression. The Clopper-Pearson test statistic was used to determine the 95% confidence intervals of the outcome variables of interest. For the outcome variable malaria, maternal age, educational level, marital status, occupation, gravidity, gestational age, IPTp-SP and LLIN use and ANC as scheduled were included in the multivariate model whereas for the outcome variable anaemia, educational level, location, malaria parasitemia and ANC as scheduled were included in the multivariate model. The model goodness of fit was assessed using the Hosmer and Lemeshow test. The model correctly classified 91.0% of the malaria cases and 66.5% of the anaemia cases. We also determined the population attributable fraction (PAF) to estimate the excess anaemia cases that can be attributed to malaria among the pregnant women studied using the category specific formula; PAF = pd[(RR_*i*_-1)/ RR_*i*_)] where *pd* = the proportion of anaemia cases exposed to malaria, and RR_*i*_ = the adjusted relative risk for exposure to malaria relative to no exposure to malaria [18]. Applying the aforementioned formula, the 95% CI of the PAF was computed using the 95% CI of the corresponding adjusted RR_*i*_ [19].

### Ethical consideration

Ethical clearance for the study was obtained from the Committee on Human Research, Publications and Ethics (CHRPE) of the School of Medical Sciences (SMS), Kwame Nkrumah University of Science and Technology, Kumasi. Permission to undertake the study at the Akatsi South District Hospital was sought and granted by the Hospital Management and the Head of the laboratory before data collection. In addition, written consent was obtained from all participants who agreed to partake in the study after they were thoroughly informed and sensitized about the study.

## Results

### Socio-demographic characteristics of the pregnant women

This study recruited 200 pregnant women. Of these, 13.5% were below 20 years while 52.0% were aged 20–29 years. The majority of the pregnant women (77.5%) had formal education below the secondary level and lived in communities outside of Akatsi (57.5%). Further, most of the pregnant women in this study were multigravida (71.0%) whereas near half (47.0%) of the were in the second trimester of gestation. Meanwhile, 62.0% of the pregnant women were primi-multiparous. At the time of this study, 83.5% of the pregnant women reported that they attended ANC as scheduled whereas 87.0% adhered to IPTp-SP prophylaxis for malaria (Table 1).

**Table 1:** Socio-demographic and clinical characteristics of the pregnant women, Akatsi South District, Ghana.

Variable	Frequency	Percentage (%)
**Overall**	200	100.00
**Maternal age (Years)**		
<20	27	13.5
20–29	104	52.0
≥30	69	34.5
**Level of education**		
Below secondary	155	77.5
Secondary and above	45	22.5
**Marital status**		
Single	40	20.0
Married	160	80.0
**Location**		
In Akatsi	85	42.5
Out Akatsi	115	57.5
**Occupation**		
Unemployed	12	6.0
Formal	89	44.5
Informal	75	37.5
Student	24	12.0
**Gravidity**		
Primigravidae	58	29.0
Multigravidae	142	71.0
**Parity**		
Nulliparaous	76	38.0
Primi-multiparous (≥ 1 Births)	124	62.0
**Gestational Period**		
First Trimester	48	24.0
Second Trimester	94	47.0
Third Trimester	58	29.0
**ANC as Scheduled**		
Yes	167	83.5
No	33	16.5
**Adhered to IPTp-SP Regimen**		
Yes	174	87.0
No	26	13.0

#### Distribution of malaria, anaemia and malaria/anaemia comorbidity stratified by maternal age

**Fig 2:**
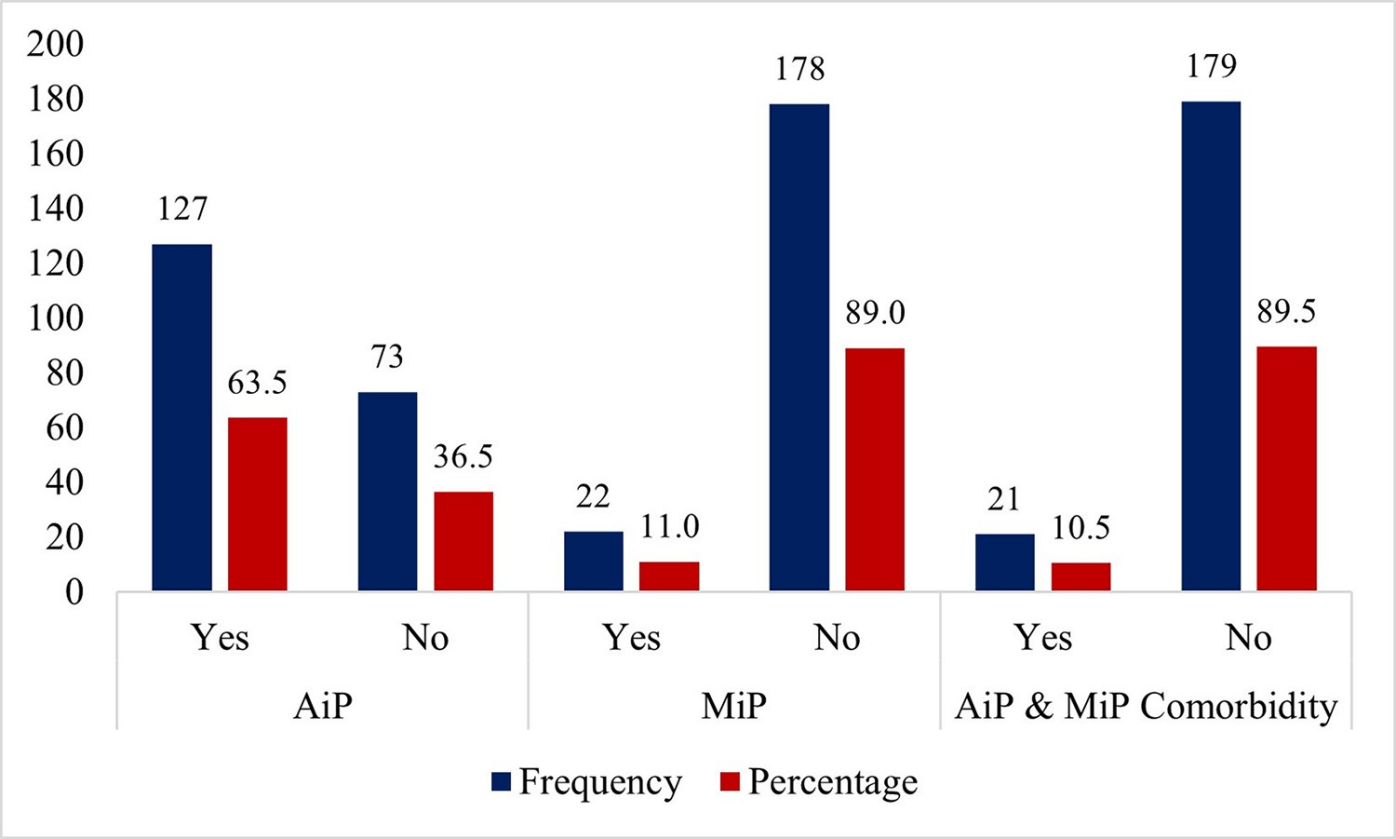
Distribution of malaria, anaemia and malaria/anaemia comorbidity stratified by maternal age.

This study recorded a MiP prevalence rate of 11.0% (96% CI:7.0–16.2). The prevalence of AiP is 63.5% (95% CI:56.4–70.2) while MiP/AiP comorbidity rate is 10.5% (95% CI:6.6–15.6) (Fig 2).

**Fig 3:**
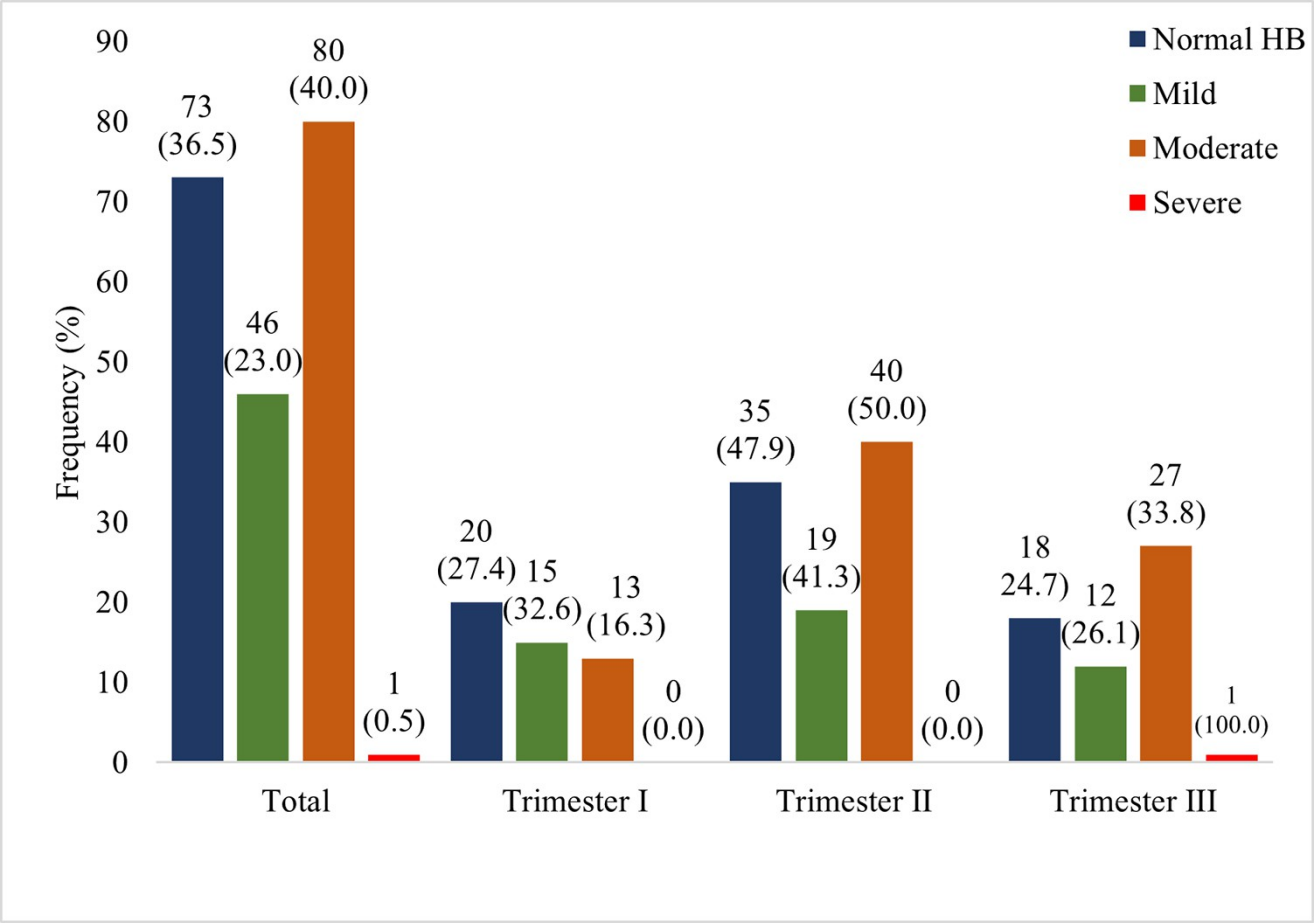
Anaemia classification stratified by gestational period. HB-Hemoglobin.

Prevalence of mild moderate and severe anaemia was 23.0%, 40.0% and 0.5% respectively. Mild anaemia (32.6%) was higher among pregnant women in their first trimester of gestation whereas moderate anaemia (50.0%) predominated among pregnant women in their second trimester of gestation. The only severe case of anaemia recorded was in a pregnant woman in her third trimester. Generally, prevalence rates of both mild and moderate anaemia rose from the first to the second trimester and then declined steeply in the third trimester of gestation (Fig 3).

#### IPTp-SP, LLIN and repellent use stratified by malaria, anaemia, and malaria/anaemia comorbidity

This study recorded 64.0% (95% CI:56.9–70.6), 90.0% (95% CI:85.0–93.8) and 42.0% (95% CI:35.1–49.2) of the pregnant women using IPTp-SP, sleeping under LLIN and using mosquito repellent respectively. As shown in Table 2 below, no cases of malaria was recorded among majority of pregnant women who used IPTp-SP (70.2%) and LLIN (94.4%). Additionally, most pregnant women who used IPTp-SP (74.0%) and slept under LLIN (95.9%) presented with normal hemoglobin concentrations. Finally, we found that no cases of MiP/AiP comorbidity was recorded among 75.0% and 95.8% of the pregnant women who used IPTp-SP and slept under LLIN respectively. Of note also is the fact that the use of mosquito repellent did not appear to have a protective function against malaria infection and MiP/AiP comorbidity. However, combined utilization of IPTp-SP, LLIN and repellent appeared to have contributed significantly to the prevention of MiP, AiP and MiP/AiP comorbidity among the study participants.

**Table 2:** IPTp-SP, LLIN and repellent use stratified by malaria, anaemia, and malaria/anaemia comorbidity.

Variables	IPTp-SP		LLIN		Repellent		IPTp-SP *LLIN*Repellent	
	Yes	No	Yes	No	Yes	No	Yes	No
**Overall**	128(64.0)	72(36.0)	180(90.0)	20(10.0)	84(42.0)	116(58.0)	50(25.0)	4(2.0)
**MiP**								
Yes	3(13.6)	19(86.4)	12(54.5)	10(45.5)	11(50.0)	11(50.0)	0(0.0)	2(9.1)
No	125(70.2)	53(29.8)	168(94.4)	10(5.6)	73(41.0)	105(59.0)	50(28.1)	2(1.1)
**AiP**								
Yes	74(58.3)	53(41.7)	110(86.6)	17(13.4)	53(41.7)	74(58.3)	24(18.9)	3(2.4)
No	54(74.0)	19(26.0)	70(95.9)	3(4.1)	31(42.5)	43(58.9)	26(35.6)	1(1.4)
**MiP & AiP Comorbidity**								
Yes	3(14.3)	18(85.7)	11(52.4)	10(47.6)	10(47.6)	11(52.4)	0(0.0)	2(9.5)
No	54(75.0)	18(25.0)	69(95.8)	3(4.2)	30(41.7)	42(58.3)	26(36.1)	0(0.0)

MiP-Malaria in Pregnancy; AiP-Anaemia in Pregnancy; Data presented as frequency and percentages in parenthesis.

#### Distribution of IPTp-SP, LLIN, and repellent use stratified by gestational period

At the time of this study, of the total number of pregnant women who reported to the hospital during the first trimester, 81.3% representing the majority slept under LLIN. Among those in their second trimester, the majority (88.3%) slept under LLIN followed closely by 69.1% on IPTp-SP. All the pregnant women who reported to the hospital during their third trimester used IPTp-SP and slept under LLIN. The rise in IPTp-SP and LLIN use from first to third trimester was statistically significant. There was no significant difference in the use of mosquito repellents over the three gestational periods (Table 3).

**Table 3:** Distribution of IPTp-SP, LLIN and repellent use stratified by gestational period.

Parameter	Total	IPTp-SP, n (%)	LLIN, n (%)	Repellent, n (%)
**Gestational Period**				
First Trimester	48	5(10.4)	39(81.3)	20(41.7)
Second Trimester	94	65(69.1)	83(88.3)	38(40.4)
Third Trimester	58	58(100.0)	58(100.0)	26(44.8)
**x**^**2**^ **for trend (p-value)**		89.34(<0.0001)	10.53(0.0011)	0.13(0.0.7225)

IPTp-SP- Intermittent Preventive Treatment during pregnancy with Sulphadoxine-pyrimethamine; LLIN- Long-Lasting Insecticide Treated Bed Nets; p-value is significant at <0.05.

#### Factors associated with malaria in pregnant women

This study revealed MiP predominance among pregnant women aged <20 years (18.5%). It was observed that most single pregnant women (22.5%) as well as primigravida pregnant women (15.5%) and pregnant women in their first trimester of gestation (18.8%) had MiP at the time of this study. MiP also predominated among pregnant women who were students (25.0%) and have had a below secondary level education (13.5%). Further, pregnant women who lived outside Akatsi township had higher MiP prevalence (12.2%) than those who lived within Akatsi (9.4%). Prevalence of MiP was also highest among pregnant women who failed to regularly attend ANC (18.2%) and those who did not adhere to the IPTp-SP malaria prophylaxis (38.5%). After adjusting for possible confounding variables, not being on IPTp-SP program posed over eleven times risk of MiP (aOR = 11.70; 95% CI:2.32–58.96; p = 0.003) compared to their counterparts on the IPTp-SP program. Similarly, pregnant women who did not sleep under LLIN were over eight times (aOR = 8.07; 95% CI:1.98–32.92; p = 0.004) at risk for MiP compared to their counterparts who used LLIN (Table 4).

**Table 4:** Factors associated with malaria in pregnant women, Akatsi South District, Ghana.

Variable	Malaria n (%)	cOR (95% CI)	p-value	aOR (95% CI)	p-value
**Maternal Age (yrs.)**					
< 20	5(18.5)	3.69(0.91–14.99)	0.068	0.54(0.02–12.46)	0.701
20–29	13(12.5)	2.32(0.72–7.44)	0.157	1.67(0.36–7.62)	0.510
≥30	4(5.8)	1		1	
**Educational Status**					
Below secondary	21(13.5)	6.90(0.90–52.76)	0.063	9.36(0.92–94.98)	0.058
Secondary and above	1(2.2)	1		1	
**Marital Status**					
Single	9(22.5)	3.28(1.29–8.35)	**0.013**	4.14(0.48–35.93)	0.198
Married	13(8.1)	1		1	
**Occupation**					
Formal	9(10.1)	1		1	
Informal	6(8.0)	0.77(0.26–2.28)	0.641	0.29(0.07–1.21)	0.090
Student	6(25.0)	2.96(0.94–9.38)	0.065	1.04(0.10–10.38)	0.971
Unemployed	1(8.3)	0.81(0.09–7.01)	0.847	1.69(0.11–25.25)	0.702
**Location**					
In Akatsi	8(9.4)	1			
Out Akatsi	14(12.2)	1.33(0.53–3.34)	0.538		
**Gravidity**					
Primigravidae	9(15.5)	1		1	
Multigravida	13(9.2)	0.55(0.22–1.36)	0.197	0.97(0.16–5.85)	0.975
**Gestational Period**					
First Trimester	9(18.8)	1		1	
Second Trimester	13(13.8)	0.69(0.28–1.72)	0.423	1.66(0.40–6.92)	0.486
Third Trimester	0(0.0)	0.04(0.00–0.63)	**0.023**	0.33(0.01–8.74)	0.506
**Preventive Method**					
***IPTp-SP***					
Yes	3(2.3)	1		1	
No	19(26.4)	14.94(4.24–52.62)	**0.000**	11.70(2.32–58.96)	**0.003**
***LLIN***					
Yes	12(6.7)	1		1	
No	10(50.0)	14.00(4.88–40.17)	**0.000**	8.07(1.98–32.92)	**0.004**
**Attended ANC as Scheduled**					
Yes	16(9.6)	1		1	
No	6(18.2)	2.10(0.75–5.84)	0.156	1.58(0.30–8.14)	0.588

IPTp-SP- Intermittent Preventive Treatment during pregnancy with Sulphadoxine-pyrimethamine; LLIN- Long-Lasting Insecticide Treated Bed Nets; ANC-Antenatal Care; cOR = Crude Odds Ratio; aOR = Adjusted Odds Ratio; CI = Confidence Interval

#### Factors associated with anaemia in pregnant women

In this study, the preponderance of the AiP cases was observed among pregnant women aged <20 years (66.7%), with a below secondary level education (67.7%). Similarly, a higher prevalence of AiP was observed among pregnant women who lived outside the Akatsi township (67.8%) and who tested positive for MiP (95.5%). Pregnant women who did not attend ANC as scheduled presented with a higher prevalence of AiP. After adjusting for possible confounding variables, being positive for malaria posed a significantly 12.10 times risk (95% CI:1.56–93.67; p = 0.017) of AiP compared to those negative for malaria whereas failure to attend ANC as scheduled posed 6.34 times risk (95% CI:1.81–22.19; p = 0.004) of AiP among the pregnant women studied (Table 5).

**Table 5:** Factors associated with anaemia in pregnant women, Akatsi South District, Ghana.

Variable	Anaemic n (%)	cOR (95%CI)	p-value	aOR (95%CI)	p-values
**Maternal Age (yrs.)**					
< 20	18(66.7)	1.29(0.50–3.27)	0.598		
20–29	67(64.4)	1.16(0.62–2.18)	0.636		
≥30	42(60.9)	1			
**Educational Status**					
Below secondary	105(67.7)	2.20(1.12–4.31)	**0.022**	1.68(0.82–3.43)	0.156
Secondary and above	22(48.9)	1		1	
**Marital Status**					
Single	23(57.5)	1			
Married	104(65.0)	1.37(0.68–2.78)	0.379		
**Occupation**					
Formal	53(59.6)	1			
Informal	50(66.7)	1.36(0.72–2.58)	0.348		
Student	16(66.7)	1.36(0.53–3.51)	0.527		
Unemployed	8(66.7)	1.36(0.38–4.85)	0.637		
**Location**					
In Akatsi	49(57.6)	1		1	
Out Akatsi	78(67.8)	1.55(0.87–2.77)	**0.140**	1.14(0.60–2.14)	0.687
**Gravidity**					
Primigravidae	36(62.1)	1			
Multigravida	91(64.1)	1.09(0.58–2.05)	0.788		
**Gestational Period**					
First Trimester	28(58.3)	1			
Second Trimester	59(62.8)	1.20(0.59–2.44)	0.608		
Third Trimester	40(69.0)	1.59(0.71–3.53)	0.257		
**Malaria Parasitemia**					
Positive	21(95.5)	14.26(1.88–108.42)	**0.010**	12.10(1.56–93.67)	**0.017**
Negative	106(59.6)	1		1	
**Attended ANC as Scheduled**					
Yes	97(58.1)	1		1	
No	30(90.9)	7.23(2.12–24.59)	**0.002**	6.34(1.81–22.19)	**0.004**

ANC-Antenatal Care; cOR = Crude Odds Ratio; aOR = Adjusted Odds Ratio; CI = Confidence Interval

## Discussion

Malaria and anaemia are two major problems of public health concern responsible for morbidity and mortality especially among pregnant women. In this study, we found an overall, 11.0%, 63.5% and 10.5% MiP, AiP and MiP/AiP comorbidity prevalence rates respectively among the pregnant women studied. Both mild and moderate anaemia predominated among pregnant women in their second trimester. We also found high utilization of IPTp-SP and LLIN by the pregnant women in the Akatsi South District while the proportion who used mosquito repellent fell below average of the total sample studied. MiP was significantly associated with non IPTp-SP and LLIN use whereas AiP was significantly associated with MiP and irregular attendance to ANC.

Varied malaria and anaemia prevalence rates have been documented in different studies within the African sub-regions. Among pregnant women in Ethiopia for example, a meta-analysis reported a pooled malaria prevalence from seven studies to be 12.72% [20]. Other studies recorded varying malaria prevalence rates either higher or lower than recorded in this study [3, 21–24]. In Ghana, MiP prevalence rates of 14.1%/13.4% by RDTs/PCR [25] and 20.4% [26], all higher than observed in this study were reported in Northern Ghana and the middle belt of Ghana respectively. The prevalence rate of malaria recorded in this study is relatively low compared to the other studies cited above although the study was conducted within the rainy season. This largely represent a good reflection of the tremendous input by relevant health authorities vis-à-vis the Ghana Health Service and District Health Directorate of the Akatsi South District in combating malaria within the district. This can be observed in the high utilization of the IPTp-SP and LLIN among the pregnant women studied which is in line with Ghana’s current malaria prevention strategy adopted from the WHO [13]. In addition, a greater percentage (87.0%) of the pregnant women reported adherence to the IPTp-SP regimen and this may also contribute to the relatively low malaria prevalence observed in this study. Notable also is that unlike reported by Anabire and colleagues where almost equal proportions of pregnant women were recruited across the three gestational periods and the highest malaria prevalence was recorded among those in their third trimester of gestation [25], the majority of the pregnant women in our study were in their second trimester of gestation followed by those in their third trimester; the two gestational periods within which the highest proportion of IPTp-SP (69.1% vs 100.0%) and LLIN (88.3% vs 100.0%) utilization was recorded in this study. This could also contribute immensely to the low malaria prevalence recorded in this study.

Meanwhile, because this study was hospital-based, the prevalence as recorded may not entirely be reflective of the actual situation on the ground. This is because issues such as preference for home delivery, non-attendance of regular ANC (observed among 16.5% of pregnant women in our study) due to hindrances such as poverty and/or access to nearer health facility among others continue to remain a challenge. Till date, non-utilization of LLIN [27] and IPTp-SP [28] continue to confront us. It is therefore imperative to intensify public education on the importance of adhering to the use of IPTp-SP and LLIN and while doing so, ensure to encourage women to regularly attend ANC at all costs for appropriate healthcare considering the significance of regular ANC attendance [29, 30].

In this study, MiP was highest among pregnant women below 20 years old. This finding is at variance with reports made among pregnant women in Hohoe municipality where malaria prevalence was highest among pregnant women aged 20–29 years [31]. Other studies reported malaria predominance among different age groups depending on the age stratification per the study design [26, 32, 33]. The cause of high MiP among pregnant women below 20 years could be because most of them (85.2%) were primigravida as against the other age groups where most of them were multigravida. Indeed, most of the malaria cases were diagnosed among the primigravida women in this study. This however did not come as a surprise since it has been well established that malaria parasitemia is often higher in primigravidae women and lower in subsequent pregnancies [34] due to lack of specific immunity to placental malaria usually acquired during previous malaria episodes [21].

The prevalence of AiP recorded in this study represents a severe public health problem [7, 9, 35]. This prevalence is higher than the crude prevalence of 26.2% reported in Kenya [36], the 19.2% in Rwanda [37], 18.0% in Northern Tanzania [9] and the 34.85% in Eastern Africa [38]. The prevalence as recorded in this study is also higher than the national prevalence of 42.4% documented in 2014 among women aged 15–49 years [39]. In Ghana, other studies among similar populations reported comparable findings [31, 40–42]. Clearly, the consistency of high AiP prevalence in Ghana cannot be underestimated just as observed by Dei-Adomakoh and colleagues some six years ago [43]. One of many contributory factors to the high AiP prevalence recorded in this study could be malaria particularly because Ghana is a malaria endemic country. However, comorbid MiP/AiP prevalence in this study is 10.5% and PAF of AiP due to MiP is 34.5% (95% CI:23.8–43.6); this implies that a chunk of the pregnant women had anaemia because of other factors aside from malaria. Though one of such factors could be iron deficiency which has been reported to account for over 100,000 maternal deaths (22% of all maternal deaths) and over 600,000 perinatal deaths globally [44], accurate cause and effect between anaemia and iron deficiency could not be established due to the deficiency of this study in assessing the recommended routine iron supplementation among the pregnant women recruited [35]. Further, this study did not review the current medical records of the pregnant women to establish the absence or otherwise of any chronic conditions capable of causing anaemia. Lastly, although helminth infections and hemoglobinopathies have been identified as important risk factors of anaemia [45, 46], stool examination and hemoglobin genotyping could not be performed during this study. The point to consider however is that the prevalence as recorded is high and a cause for concern. Further studies geared towards establishing the major causative factors of anaemia among pregnant women in the Akatsi South District are therefore indicated.

In this study, the prevalence of AiP was highest among pregnant women below 20 years old, akin to that reported by Fondjo and colleagues [7] but contrary to findings in other parts of Ghana [31, 42]. Other studies recorded anaemia predominance among varied age categorizations [32, 37, 40, 41, 47]. The higher AiP prevalence observed among pregnant women below 20 years could be due to malnutrition such as iron, vitamin B12/folate deficiency etc. among this age group. As stated earlier however, accurate cause and effect between these factors and anaemia could not be established in our findings due to assessment failure of these factors among the pregnant women in this study. Meanwhile, considering the high prevalence of MiP among pregnant women in this age category (18.5%), the hemolytic effect of malaria could be blamed for the predominant AiP prevalence observed among this age group. It is also possible that environmental, and physiological factors such as rate of hemopoiesis, psychological and emotional stability including mental peace, effects of poverty, hormonal imbalances, etc., all during pregnancy could directly or indirectly impact negatively on the blood levels of these pregnant women. This becomes important considering the young age of these pregnant women and the fact that most of them got pregnant unprepared.

Compared to findings by Fondjo and colleagues who documented mild, moderate and severe anaemia prevalence rates of 35.7%, 6.1% and 0.6% respectively [7], our study found lower mild anaemia prevalence (23.0%), higher moderate anaemia prevalence (40.0%) and comparable severe anaemia prevalence rate of 0.5% among the pregnant women. Both mild and moderate anaemia was found highest among pregnant women in their second trimester similar to findings by Dei-Adomakoh and colleagues [43] who found high anaemia prevalence (70.0%) among pregnant women in their second trimester. Hemoglobin concentration often declines until birth and the cause of anaemia is relative to plasma volume and red cell mass with both increasing during the course of pregnancy. Nonetheless, the plasma volume increases proportionately higher than the red cell mass. Perhaps a high rate of increase in plasma volume during the second trimester akin to that observed by Aguree and Gernand [48] may therefore account for the high mild and moderate anaemia prevalence observed during that period.

Whereas this study recorded high utilization of both IPTp-SP and LLIN among the pregnant women studied, lower rates of utilization has been documented elsewhere [27, 49]. In fact, in Cameroon, of the 81.0% pregnant women who owned bed nets via antenatal visits, only 42.7% adhered to sleeping under them [23]. For IPTp-SP, low consumption rate (42.4%) of 3 doses was reported in Northern Ghana [50] compared to the high level of consumption of 3 doses (64.5%) documented in a similar study in Southern Ghana [28]. The proportion of pregnant women who utilized IPTp-SP in this study (64.0%) is higher than the 29.5% (95% CI = 28.2–30.5) documented by Yaya and colleagues [51]. However, the proportion of IPTp-SP utilization documented for Ghana (60%, 95% CI = 57.1–62.8) [51] in their study is akin to that observed in this study. Though our study failed to assess the number of doses of IPTp-SP taken by the pregnant women and by extension the number of doses taken per each gestational period, the use of IPTp-SP as well as LLIN significantly increased with advancement in gestational age which we believe can be attributed to the high attendance of scheduled ANC (83.5%) as supported by other findings [29, 52]. This great achievement coupled with testing and treatment will help advance the eradication of malaria related morbidity and mortality among pregnant women in the Akatsi South District and by extension among pregnant women in Ghana.

This study revealed that pregnant women who were not on the IPTp-SP program (OR = 11.70) and did not sleep under LLIN (OR = 8.07) were at significant risk of suffering from malaria. Fondjo and colleagues reported moderately and severely self-reporting mosquito exposure and non-repellent use as significant independent contributing factors to malaria [7]. In Hohoe Municipality, Ghana, women who were anaemic and those who visited focused-ANC 5–8 times were 1.70 and 2.08 times more likely to have malaria respectively [31]. These findings suggest that different populations are often affected by different risk factors. Population-based risk assessment for every community could therefore be a steppingstone into supporting the fight against malaria among pregnant women. Nonetheless, there is the need to scale up the distribution of LLINs and administration of IPTp-SP among pregnant women in the spirit of ensuring that all pregnant women are protected from malaria.

We also found that malaria as well as irregular ANC attendance produced a 12.10 and 6.34 likelihood of anaemia among the pregnant women compared to their counterparts negative for malaria and who attended ANC as scheduled. In Northern Tanzania, the clinic of recruitment and low level of education of the women were the independent contributing factors associated with anaemia during pregnancy [9] while other studies reported other risk factors [40–42]. Exposure to different environmental conditions yields varying risk factors for different health issues including anaemia. Hence, just as stated relative to malaria, population-specific risk assessments will contribute immensely to identifying the major risk factors the pregnant women are exposed to help contribute to the fight against anaemia among pregnant women in the Akatsi South District of Ghana.

## Conclusion

The prevalence rate of MiP is relatively low among pregnant women in the Akatsi South District compared to other findings cited in Ghana. From our observation, the high utilization of IPTp-SP and LLIN among the pregnant women played a significant role in the low malaria prevalence observed even though the study was conducted during the rainy season where a surge in malaria cases is often recorded. Scaleup of IPT-SP supplementation and LLIN distribution will help to further reduce the prevalence of MiP within the district. The prevalence of AiP on the other hand is very high with a chunk of the cases unrelated to malaria as a probable cause. Our study did not assess the regular WHO recommended iron supplementation among pregnant women as well as other conditions capable of causing anaemia such as hemoglobinopathies and helminthiasis. As a result, an accurate cause and effect relationship between these factors and anaemia could not be established. Further studies to enumerate the major factors responsible for anaemia among pregnant women in the Akatsi South District are hence recommended to help inform health authorities on the appropriate strategies for combating the hiking anaemia cases among pregnant women in the district. Not being on the IPTp-SP program and not sleeping under LLIN posed a significantly increased risk of malaria among the pregnant women whereas malaria as well as irregular ANC attendance produced an increased likelihood of anaemia among pregnant women. We therefore recommend regular and intensified education on adherence to IPTp-SP intake, LLIN use and regular ANC attendance to help combat MiP and AiP in the Akatsi South District.

## Supporting information

S1 File Research questionnaire. (DOCX)
