# Supplementary material for: Prevalence and risk factors of malaria and anaemia and the impact of preventive methods among pregnant women: A case study at the Akatsi South District in Ghana
Source: PLoS One. 2022 Jul 25;17(7):e0271211. doi: 10.1371/journal.pone.0271211 (PMC9312417; doi:10.1371/journal.pone.0271211)
Supplement: S1 File — (DOCX) [file pone.0271211.s001.docx]

**QUESTIONNAIRE**

**Prevalence and risk factors of malaria and** **anaemia and the impact of preventive methods among pregnant women: A case study at the Akatsi South District in Ghana**

| I have given my consent to take part in this study | | | | YES | |  | | NO |  |
| --- | --- | --- | --- | --- | --- | --- | --- | --- | --- |
| Code: ……………………… | | | | | | Date_____/_____/ 2016 | | | |
| 1. Sociodemographic information of respondents | | | | | | | | | |
| S/N | Parameters/Questions |  |  | | | Responds | | | |
|  |  | Please tick appropriately as applied to you | | | | | | | |
| 1 | Maternal age | ………………………………... | | | | | | | |
| 2 | Occupation | ……………………………………………………………………… | | | | | | | |
| 3 | Marital status | Single |  | | | Married | |  | |
|  |  | Cohabiting |  | | |  | | | |
| 4 | Level of education | No education |  | | | Basic | |  | |
|  |  | Secondary |  | | | Tertiary | |  | |
| 5 | Residence (to be classified into **In Akatsi** and **Out Akatsi** by the research team) | ……………………………………………………………………… | | | | | | | |
| 1. Obstetric characteristics of respondents | | | | | | | | | |
| 6 | Number of pregnancies (Gravidity) | ……………………………………………………………………… | | | | | | | |
| 7 | Number of births (Parity) | ……………………………………………………………………… | | | | | | | |
| 8 | Gestational period | Trimester I |  | | | Trimester II | |  | |
|  |  | Trimester III |  | | |  | | | |
| 9 | Attend ANC as scheduled | Yes | |  | No | |  | | |
| 1. **Assessment of malaria prevention programmes** | | | | | | | | | |
|  |  | | | | | Yes | | No | |
| 10 | Are you on IPTp-SP? | | | | |  | |  | |
| 12 | Do you adhere to the IPTp-SP regimen given you at the ANC? | | | | |  | |  | |
| 14 | Do you use long-lasting insecticide treated bed nets (LLIN)? | | | | |  | |  | |
| 16 | Do you use mosquito repellent? | | | | |  | |  | |
| 17 | Do you use mosquito use mosquito sprays? | | | | |  | |  | |
| 18 | Do you use mosquito use mosquito coil? | | | | |  | |  | |
| 19 | Do you use mosquito use mosquito bite prevention creams? | | | | |  | |  | |
| 1. **Laboratory investigations** | | | | | | | | | |
| 20 | Haemoglobin Concentration (g/dL): ……………………………………………………………. | | | | | | | | |
| 21 | Blood film for malaria parasite results: …………………………………………………………. | | | | | | | | |
